# Asymptomatic heterotopic pancreas in Meckel’s diverticulum: a case report and review of the literature

**DOI:** 10.1186/s13256-015-0576-x

**Published:** 2015-05-09

**Authors:** Alfredas Kilius, Narimantas Evaldas Samalavicius, Donatas Danys, Gytis Zaldokas, Dmitrij Seinin

**Affiliations:** 1 Center of Oncosurgery, National Cancer Institute, Vilnius University, 1 Santariskiu Street, LT-08406 Vilnius, Lithuania; 2 Center of Oncosurgery, National Cancer Institute, Clinic of Internal Diseases, Family Medicine and Oncology of Medical Faculty, Vilnius University, 1 Santariskiu Street, LT-08406 Vilnius, Lithuania; 3 Vilnius University, Medical Faculty, Vilnius, Lithuania; 4 National Center of Pathology, P. Baublio 5, Vilnius, LT-08406 Lithuania

**Keywords:** Heterotopic pancreas, Meckel’s diverticulum, Surgery

## Abstract

**Introduction:**

Heterotopic pancreas is defined as pancreatic tissue without a real anatomical or vascular connection to the pancreas. It can be found in the stomach, duodenum, jejunum, ileum, Meckel’s diverticulum, colon gall bladder, umbilicus, fallopian tube, mediastinum, spleen and liver. Complications of heterotopic pancreas are inflammation, bleeding, obstruction, malignant transformation, carcinoid syndrome, jejunojejunal intussusception and ileus, but it is usually asymptomatic and diagnosed only during examinations for other diseases.

**Case presentation:**

An 81-year-old Lithuanian woman was diagnosed with caecal cancer and had undergone elective surgery. A right hemicolectomy was performed and a Meckel’s diverticulum was observed and excised. Histological results showed a poorly differentiated G3 adenocarcinoma of her large intestine and heterotopic pancreas tissue in the Meckel’s diverticulum and mesenteric adipose tissue.

**Conclusions:**

Asymptomatic heterotopic pancreas is rarely diagnosed, and usually found incidentally during surgical or diagnostic interventions. Although it has no symptoms, heterotopic pancreas found during surgical procedures should be excised.

## Introduction

Heterotopic pancreas (HP) is defined as pancreatic tissue without real anatomical or vascular connection to the pancreas and was first reported by Jean-Schultz in 1729 [1,2]. The incidence of HP is 0.5 to 13.0% in autopsy studies [3]. The most common sites of HP are the stomach, duodenum, and jejunum, but it can be also found in the ileum, Meckel’s diverticulum (MD), colon, gall bladder, umbilicus, fallopian tube, mediastinum, spleen, and liver [4]. HP can cause complications including inflammation, ulceration, chemical irritation, bleeding, obstruction, malignant transformation, jejunojejunal intussusception and ileus [5-8]. However, most patients with ectopic pancreas are asymptomatic and diagnosis is usually confirmed during a radiological examination or endoscopy of the digestive tract or during surgical explorations motivated by other diseases [9]. On microscopic examination, ectopic pancreas is composed of varying amounts of pancreatic ducts, acini and islets of Langerhans [7]. Heinrich’s criteria are used to classify HP: type 1 contains cells of exocrine glands, excretory ducts and islets of Langerhans; type 2 contains only excretory glands and excretory ducts; type 3 contains only excretory ducts [10]. We report an unusual case of HP found in MD.

## Case presentation

**Figure 1:**
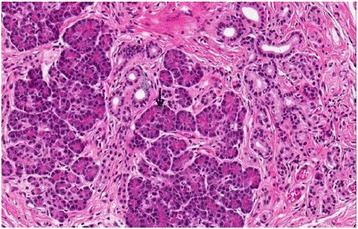
Histological section (hematoxylin and eosin ×200) of the small intestine. The figure shows the presence of pancreatic tissue (black arrow – pancreatic acini) in the small intestine and a muscular layer of Meckel’s diverticulum with cystic dilatation of the ducts (blue arrow).

An 81-year-old Lithuanian woman was tested for faecal occult blood during preventive colorectal cancer screening. Test results were positive and she underwent a colonoscopy. The colonoscopy revealed a tumour in her caecum. Abdominal and thoracic computed tomography (CT) scanning revealed no metastasis. She then underwent elective surgery. During the operation, a 6×5cm tumour was found in her caecum. Her MD was found 40cm from the hepatoduodenal ligament [6]. A right hemicolectomy was performed to excise the MD from her jejunal loop. After the operation, she underwent chemotherapy. There were no complications related to the surgery. Histological results showed a poorly differentiated G3 adenocarcinoma of the caecum. In her small intestine a muscular layer of excised MD and mesenteric adipose tissue was found, which was pancreatic tissue morphologically. Microscopic analysis revealed pancreatic tissue without islets of Langerhans (Figure 1) in the small intestine and mesenteric adipose tissue. Moreover, in the HP tissue, dilatation of the pancreatic ducts was observed.

## Discussion

The aetiology of HP, otherwise known as “aberrant”, “ectopic”, or “accessory” pancreas, is still unclear, but it is believed to arise embryonically during rotation of the foregut and fusion of the dorsal and ventral pancreatic buds. Some tissue separates from the pancreas and develops into HP in any portion of the alimentary system. Another best known theory is based on the pancreatic metaplasia of endodermal tissue [4,5]. Because of the proximity of the embryonic pancreatic primordial buds and the foregut during development, it is not surprising that 70 to 90% of ectopic pancreas occurs in the upper gastrointestinal system [9]. MD is the second most common site of heterotopic tissue; the stomach is the most common site (50 to 60% of cases) while 5 to 16% of cases occur in the pancreas [9]. Surgical excision is the first and best choice of treatment because medical treatment is not effective [5]. However, the treatment of HP depends on the patient. Asymptomatic patients with a positive diagnosis must remain under medical supervision and be reviewed periodically. Symptomatic patients without complications should have their lesions excised, preferably by local resection. In sites accessible by fibrescope, endoscopic removal may be performed. HP found during surgical procedures motivated by other diseases, as in our case, should be excised and submitted for a frozen section study whenever necessary, thus avoiding possible complications and the need for reoperation. In cases of lesions associated with bleeding, obstruction or suspicion of malignancy, an appropriate surgical approach is undertaken [9]. HP can be eliminated by simple excision, as multiple lesions are unusual [11]. The preoperative diagnosis of HP in the small intestine is difficult. Symptoms depend on the size of lesion and involvement of mucosa. On clinical examination, significant lesions are greater than 1.5cm [7,9]. Most cases represent HP in a MD with gastrointestinal bleeding (Table 1), but in our case it was asymptomatic. Because HP can be found submucosally and the most common submucosal tumour is gastrointestinal stroma tumour (GIST), HP can frequently be mistaken as GIST or leiomyoma at endoscopy, ultrasonography or CT scanning [12]. In our case, HP was mistaken for metastasis of the caecal tumour. The definitive diagnosis is confirmed by pathological examination after the resection (Table 2). However, endoscopic ultrasound findings are often associated with these lesions: a diameter larger than 4cm, poorly defined margins, cystic spaces and internal echogenic foci, and adjacent lymphadenopathy and rapid growth [13]. Although, pathological examination is the most accurate diagnostic method for diagnosis of ectopic pancreas, most biopsies are inconclusive, because it is difficult to take adequate tissue samples. MD can be diagnosed with ^99m^technetium pertechnetate scintigraphy, which detects ectopic gastric mucosa in MD. However, the diagnostic accuracy of this method is 46% in adults. Abdominal ultrasound, X-ray and CT usually give nonspecific findings. MD can be detected by capsule endoscopy or double-balloon enteroscopy. The diagnosis of MD with HP is difficult [6,14]. Management of incidentally found MD is controversial. Some authors suggest that indications for diverticulectomy should be based on intraoperative findings. Wide-mouthed, thin-walled diverticula without bands could be left undisturbed, whereas thickened, narrow-based diverticula should be resected. However, there are no definite anatomic criteria to predict the probability of future complications. According to the literature, the benefits of incidental diverticulectomy outweigh its attending morbidity and mortality [15]. In our case, MD was resected because ectopy of the pancreatic tissue was considered a metastasis of the caecal tumour.

**Table 1:** **Comparison of heterotopic pancreas found in Meckel’s diverticulum**

**Authors and year**	**Symptoms**	**Complications**	**Histological view**	**Treatment**
Yang and Guo; 2013 [6]	Bloody stools, dizziness, asthenia	Gastrointestinal bleeding	Acini, ducts, islets	Resection of diverticulum
Kopáčová *et al.*; 2010 [16]	Melena	Gastrointestinal bleeding	Acini, ducts, islets	Resection of diverticulum and ileum
Zarand *et al.*; 2011 [17]	Abdominal pain, nausea	Inflammation of heterotopic pancreas tissue	Acini, ducts	Resection of diverticulum
Xiao *et al.*; 2009 [18]	Bloody stools, abdominal pain	Gastrointestinal bleeding	Acini, ducts	Resection of diverticulum and ileum
Yang *et al.*; 2011 [14]	Bloody stools, dizziness, weakness	Gastrointestinal bleeding	*Data not presented*	Resection of ileum with diverticulum
Baysoy *et al.*; 2010 [19]	Haematochezia, melena	Gastrointestinal bleeding	Acini	Resection of diverticulum

**Table 2:** **Comparison of heterotopic pancreas found in different locations**

**Authors and year**	**Localisation of heterotopic pancreas**	**Symptoms**	**Complications**	**Diagnostic methods**
Saka *et al.*; 2009 [20]	Jejunum	Bloody stools	Gastrointestinal bleeding, jejunal obstruction	Upper gastrointestinal contrast, immunohistochemical, histopathological examination
Yang and Guo; 2013 [6]	Meckel’s diverticulum	Bloody stools, dizziness, asthenia	Gastrointestinal bleeding	Laparotomy, histopathological examination
Kopáčová *et al.*; 2010 [16]	Meckel’s diverticulum	Melena	Gastrointestinal bleeding	Enteroclysis, intraoperative enteroscopy, histopathological examination
Rana *et al.*; 2009 [21]	Jejunum	Abdominal pain	No complications	Gastrointestinal endoscopy, CECT, histopathological examination
Gunjača *et al.*; 2010 [7]	Duodenum, stomach	Abdominal pain	Duodenal perforation, inflammation of HP tissue	Ultrasonography, upper endoscopy, CT, histopathological examination
Okamoto *et al.*; 2014 [5]	Jejunum	Hearing loss	Neoplasm	During surgery, histopathological examination
Lee *et al.*; 2012 [22]	Jejunum	Melena, haematochezia, dizziness	No complications	Capsule endoscopy, histopathological examination
Zarand *et al.*; 2011 [17]	Meckel’s diverticulum	Abdominal pain, nausea	Inflammation of HP tissue	During surgery, histopathological examination
Xiao *et al.*; 2009 [18]	Meckel’s diverticulum	Bloody stools, abdominal pain	Gastrointestinal bleeding	Capsule endoscopy, intraoperative endoscopy, histopathological examination
Yang *et al.*; 2011 [14]	Meckel’s diverticulum	Bloody stools, dizziness, weakness	Gastrointestinal bleeding	CECT, laparotomy, histopathological examination
Trifan *et al.*; 2012 [23]	Stomach	Abdominal pain, nausea, vomiting	Gastric outlet obstruction	Upper gastrointestinal endoscopy, abdominal ultrasound, histopathological examination
Christodoulidis *et al.*; 2007 [24]	Stomach	Abdominal pain, nausea, vomiting	No complications	EGD, laparotomy, histopathological examination
Guimarães *et al.*; 2013 [25]	Stomach	Abdominal pain	No complications	Abdominal CT and MRI, histopathological examination
Hirasaki *et al.*; 2009 [8]	Jejunum	Abdominal pain	Jejunojejunal intussusception, ileus	Abdominal CT, histopathological examination

Abbreviations: CECT, contrast-enhanced computed tomography; CT, computed tomography; EGD, esophagogastroduodenoscopy; HP, heterotopic pancreas; MRI, magnetic resonance imaging.

## Conclusions

If HP is asymptomatic, as in our case, diagnosis is unlikely without surgical intervention. Surgical interventions are usually performed in other diseases. Because of its complications and chance of malignant transformation, HP found during surgical procedure should be excised.

## Consent

Written informed consent was obtained from the patient for publication of this case report and accompanying images. A copy of the written consent is available for review by the Editor-in-Chief of this journal.

## Abbreviations

CT: Computed tomography
GIST: Gastrointestinal stroma tumour
HP: Heterotopic pancreas
MD: Meckel’s diverticulum

## References

[1] Cano DA, Hebrok M, Zenker M. Pancreatic development and diseases. Gastroenterology. 2007;132:745–62. 10.1053/j.gastro.2006.12.054 17258745

[2] Jiang LX, Xu J, Wang XW, Zhou FR, Gao W, Yu GH, et al. Gastric outlet obstruction caused by heterotopic pancreas: A case report and a quick review. World J Gastroenterol. 2008;14:6757–9. 10.3748/wjg.14.6757 PMC2773325 19034986

[3] Song DE, Kwon Y, Kim KR, Oh ST, Kim JS. Adenocarcinoma arising in gastric heterotopic pancreas: a case report. J Korean Med Sci. 2004;19(1):145–8. 10.3346/jkms.2004.19.1.145 PMC2822253 14966359

[4] Tanaka K, Tsunoda T, Eto T, Yamada M, Matsuo S, Izawa K. Diagnosis and management of heterotopic pancreas. Int Surg. 1993;78:32–5. 8473080

[5] Okamoto H, Fujishima F, Ishida K, Tsuchida K, Shimizu T, Goto H, et al. Intraductal papillary mucinous neoplasm originating from a jejunal heterotopic pancreas: report of a case. Surg Today. 2014;44(2):349–53. 10.1007/s00595-012-0486-0 23325495

[6] Yang X, Guo K. Massive lower gastrointestinal bleeding from Meckel’s diverticulum with heterotopic pancreas: case report and a brief review of the literature. JOP. 2013;14(3):269–72. 10.6092/1590-8577/1407 23669477

[7] Gunjača I, Mlinac-Lucijanic M, Pavlovic A, Gunjača M. Inflammation of ectopic pancreatic tissue as unusual case of duodenal perforation – a case report. Antropol. 2010;3:1119–22. 20977114

[8] Hirasaki S, Kubo M, Inoue A, Miyake Y, Oshiro H. Jejunal small ectopic pancreas developing into jejunojejunal intussusception: A rare cause of ileus. World J Gastroenterol. 2009;15(31):3954–6. ISSN 1007–9327. 10.3748/wjg.15.3954 PMC2731263 19701981

[9] Bromberg SH, Neto CC, Fernando A, Borger A, Franco MIF, Franca LCM, et al. Pancreatic heterotopias: clinicopathological analysis of 18 patients. Rev Col Bras Cir. 2010;37(6):413–9. 10.1590/s0100-69912010000600007 21340256

[10] Heinrich H. Ein Beitrag zur Histologie des sogen akzessorischen Pankreas. Virchows Arch Path Anat Physiol. 1909;198:392–401.

[11] Hamada Y, Yonekura Y, Tanano A, Takada K, Kato Y, Sato M, et al. Isolated heterotopic pancreas causing intussusception. Eur J Pediatr Surg. 2000;10:197–200. 10.1055/s-2008-1072357 10982052

[12] Kim JY, Lee JM, Kim KW, Park HS, Choi JY, Kim SH, et al. Ectopic pancreas: CT findings with emphasis on differentiation from small gastrointestinal stromal tumor and leiomyoma. Radiology. 2009;252(1):92–100. 10.1148/radiol.2521081441 19561251

[13] Shen EF, Arnott ID, Plevris J, Penman ID. Endoscopic ultrasonography in the diagnosis and management of suspected upper gastrointestinal submucosal tumours. Br J Surg. 2002;89(2):231–5. 10.1046/j.0007-1323.2001.02002.x 11856140

[14] Yang J, Sun L, Wang X, Dai N. Massive gastrointestinal bleeding from Meckel diverticulum with ectopic pancreatic tissue. Chin Med J. 2011;124(4):631–3. 21362295

[15] Yahchouchy EK, Marano AF, Etienne FJC, Fingerhut AL. Meckel’s diverticulum. J Am Coll Surg. 2001;192(5):658–62. 10.1016/s1072-7515(01)00817-1 11333103

[16] Kopáčová M, Vykouřil L, Vacek Z, Tyčová V, Bártová J, Rejchrt S, et al. Inverted Meckel’s diverticulum with ectopic pancreatic tissue as a source of severe gastrointestinal bleeding. J Gastrointest Surg. 2010;14:578–81. 10.1007/s11605-009-0838-2 PMC2820663 19274480

[17] Zarand A, Bajtai A, Baranyai Z, Dede K, Jakab F. Inflammation of ectopic pancreatic tissue in a Meckel’s diverticulum causing acute abdominal symptoms: a case report and review of the literature. Int J Surg Pathol. 2011;19(3):359–63. 10.1177/1066896909331995 19223375

[18] Xiao WD, Chen W, Yang H. Heterotopic pancreas within Meckel’s diverticulum with obscure then massive gastrointestinal bleeding in a 12-year-old child: case report and review of the literature. J Int Med Res. 2009;37:967–72. 10.1177/147323000903700343 19589283

[19] Baysoy G, Balamtekin N, Uslu N, Karavelioğlu A, Talim B, Özen H. Double heterotopic pancreas and Meckel’s diverticulum in a child: do they have common origin? Turk J Pediatr. 2010;52:336–8. 20718198

[20] Saka R, Gomi A, Sugiyama A, Ohashi Y, Ohike N, Shiokawa A, et al. Ectopic pancreas as a cause of jejunal obstruction in a neonate. J Pediatr Surg. 2009;44:856–8. 10.1016/j.jpedsurg.2009.01.013 19361655

[21] Rana SS, Bhasin DK, Nada R, Gupta R, Singh K. Heterotopic pancreas in the jejunum presenting a submucosal lesion on endoscopy. J Pancreas. 2009;10(4):419–20. 19581747

[22] Lee MJ, Chang JH, Maeng IH, Park JY, Im YS, Kim TH, et al. Ectopic pancreas bleeding in the jejunum revealed by capsule endoscopy. Clin Endosc. 2012;45:194–7. 10.5946/ce.2012.45.3.194 PMC3429734 22977800

[23] Trifan A, Târcoveanu E, Danciu M, Huţanaşu C, Cojocariu C, Stanciu C. Gastric heterotopic pancreas: an unusual case review of the literature. J Gastrointestin Liver Dis. 2012;21(2):209–12. 22720312

[24] Christodoulidis G, Zacharoulis D, Barbanis S, Katsogridakis E, Hatzitheofilou K. Heterotopic pancreas in the stomach: a case report and literature review. World J Gastroenterol. 2007;13(45):6098–100. 10.3748/wjg.v13.45.6098 PMC4250899 18023108

[25] Guimarães M, Rodrigues P, Gonçalves G, Nora M, Monteiro MP. Heterotopic pancreas in excluded stomach diagnosed after gastric bypass surgery. BMC Surg. 2013;13:56. 10.1186/1471-2482-13-56 PMC4222587 24267291

